# Healthcare-associated fungal outbreaks: New and uncommon species, New molecular tools for investigation and prevention

**DOI:** 10.1186/s13756-018-0338-9

**Published:** 2018-03-27

**Authors:** Marie-Elisabeth Bougnoux, Sophie Brun, Jean-Ralph Zahar

**Affiliations:** 1 Unité de Parasitologie-Mycologie, Service de Microbiologie clinique, Hôpital Necker Enfants-Malades, Assistance Publique - Hôpitaux de Paris (APHP), Paris, France; 2 Université Paris Descartes, Sorbonne Paris-Cité, Paris, France; 3 Département Mycologie, Institut Pasteur, Unité Biologie et Pathogénicité Fongiques, Paris, France; 4 Service de Parasitologie-Mycologie, Hôpital Avicenne, Groupe Hospitalier Paris-Seine-Saint-Denis, Assistance Publique - Hôpitaux de Paris (APHP), Bobigny, France; 5 Université Paris 13, Bobigny, France; 6 Département de microbiologie clinique, unité de contrôle et de prévention du risque infectieux, Hôpital Avicenne, Groupe Hospitalier Paris-Seine-Saint-Denis, Assistance Publique - Hôpitaux de Paris (APHP), Bobigny, France; 7 IAME, UMR 1137, Université Paris 13, Sorbonne Paris Cité, Paris, France

**Keywords:** Healthcare-associated fungal outbreak, *Candida auris*, *Exserohilum rostratum*, *Sarocladium kiliense*, *Saprochaete clavata*, Prevention, Whole genome sequencing

## Abstract

Outbreaks of healthcare-associated fungal infections have repeatedly been described over recent years, often caused by new or uncommon species. *Candida auris*, a recently described multidrug-resistant yeast species, is certainly the most worrisome species having caused several severe healthcare outbreaks of invasive infections, on four continents. Also, large nosocomial outbreaks due to uncommon fungal species such as *Exserohilum rostratum* and *Sarocladium kiliense*, were both linked to contamination of medical products, however the source of another outbreak, caused by *Saprochaete clavata*, remains unresolved. Furthermore, these outbreaks identified new populations under threat in addition to those commonly at risk for invasive fungal infections, such as immunosuppressed and intensive care unit patients. All of these outbreaks have highlighted the usefulness of a high level of awareness, rapid diagnostic methods, and new molecular typing tools such as Whole Genome Sequencing (WGS), prompt investigation and aggressive interventions, including notification of public health agencies.

This review summarizes the epidemiological and clinical data of the majority of healthcare-associated outbreaks reported over the last 6 years caused by uncommon or new fungal pathogens, as well as the contribution of WGS as support to investigate the source of infection and the most frequent control measures used.

## Background

Until the last decades, only a small number of fungal pathogens such as *Aspergillus, Candida*, *Pneumocystis jirovecii*, and Mucorales have been involved in healthcare-associated fungal outbreaks [1–3]. Furthermore, these outbreaks were limited to well-defined at-risk populations. Indeed, neutropenic patients, including patients treated for acute leukemia, severely ill patients during their stay in intensive care unit (ICU), and neonates hospitalized in neonatal ICU, were the main populations that suffered from these severe infections [4]. The occurrence of sporadic episodes related to other fungal species and other less immunocompromised populations remained a rare phenomenon.

Nevertheless, several recent reports have highlighted the emergence of new healthcare-associated outbreak phenomena due to fungal species previously unknown or uncommon in clinical practice. Interestingly, these fungal outbreaks affected both weak patients and healthy individuals.

Thus, a new species of *Candida*, *Candida auris*, has been recently identified and is now considered a notorious healthcare-associated yeast causing invasive infections with high treatment rate failures [5]. Diversely, during the past 6 years, rarely isolated fungi have been involved in healthcare-associated outbreaks; among these, *Exserohilum rostratum* and *Sarocladium kiliense* were related to contamination of medical products and represented a high public health threat. Also, others healthcare-associated outbreaks due to uncommon fungal species, such as *Saprochaete clavata,* are still unresolved as no source of contamination has yet been documented.

Increased vigilance and usage of advanced technologies are needed to rapidly identify the likely sources of these infections in order to efficiently guide epidemiological investigations and initiate appropriate control measures.

Since many publications have so far been interested in classical fungal infections, we aim to decipher the most notable healthcare-associated outbreaks caused in the last 6 years by uncommon or new fungal pathogens (summarized in Table 1). In order to highlight the most recent insights related to these outbreaks, we focused on (i) the epidemiological, clinical, and microbiological data, (ii) the contribution of the WGS typing method for outbreak investigation, and (iii) the recommendations to manage cases and control transmission of these uncommon fungi in healthcare facilities.

**Table 1:** Healthcare-associated outbreaks due to four new or uncommon fungal pathogens

Species	Year	Country	N° of cases	Infection sites	At risk population	Source	Main preventive measures	References
*Candida auris*	Since 2011	Four continents	> 500	IFI and colonization	Risk of invasive candidiasis	Human and environmental surfaces	Improve hand hygiene Contact isolation Improve environmental desinfection	[3–14, 36–50]
*Saprochaete clavata* (formerly *Geotrichum clavatum*)	Sept 2011-Oct 2012 Oct-Dec 2014	France Italy	30 3	Blood and colonization Spleen, liver, lung	Hematological malignancies Hematological malignancies	Unknown Unknown	Define and identify at risk population Search for a common source	[15–17]
*Sarocladium kiliense* (formerly *Acremonium kiliense*)	June 2013-Jan 2014 Nov 2013 March-May 2011	Chile (8 hospitals in Santiago) Colombia Greece	67 16 3	Blood Blood Blood	Chemotherapy Chemotherapy Stem cell transplantation	Antinausea ondansetron (company A) Ondansetron (company A) CVC	Recall of all ondansetron lots of the company A	[21–23]
*Exserohilum rostratum*	Sept 2012-Oct 2013	USA (20 states)	751	Paraspinal/spinalMeninges Peripheral joint	Epidural/paraspinal injection of MPA	MPA (compounded drug)	Recall of the 3 contaminated lots	[24–28, 33–35]

*CVC* Central venous catheter, *MPA* Methylprednisolone acetate

## Main text

### Epidemiology and clinical presentation of recently reported healthcare-associated fungal outbreaks due to New or uncommon fungal species

#### Candida auris

Over the past 6 years, *Candida auris*, a multidrug-resistant (MDR) *Candida* species, has emerged on four continents as a yeast associated with hospital outbreaks with high rates of clinical treatment failure. *C. auris* causes invasive infections in severely compromised patients, is mostly resistant to fluconazole and exhibits variable susceptibility to other azoles, amphotericin B, and echinocandins. Unlike other *Candida* species, *C. auris* seems to have a high propensity for patient-to-patient transmission in healthcare settings, possibly related to environmental contamination, or transient person or device colonization [6].

The serious threat posed by *C. auris* prompted the US Center for Disease prevention and Control (CDC) to issue an alert in June 2016 addressed to healthcare facilities on notifying cases of *C. auris* to local or public health authorities [7]. Public Health England also published guidelines for actively identifying and reporting *C. auris* to prevent its nosocomial transmission [8] and the European CDC (ECDC) [9] issued a health alert for strict vigilance of *C. auris cases*.

*C. auris* was first described in 2009 from a patient’s ear swab in Japan [10]. The first three cases of nosocomial bloodstream infections related to *C. auris* were described in South Korea in 2011 [11]. These three cases highlighted the feature of persistent candidemia despite fluconazole and amphotericin B therapy. Thereafter, in a short span of 6 years, cases of *C. auris* fungaemia and deep-seated infections have been reported from hospitals across four continents [9]. *C. auris* is probably underdiagnosed as this yeast is often phenotypically misidentified as *Candida haemulonii*, *Candida famata*, or *Candida sake* by commercial biochemical identification systems [12, 13].

*C. auris* causes infections in patients, ranging from neonates to elderly, with well-recognized risk factors for invasive candidiasis. Indeed, among the 41 *C. auris* deep infections observed during 2012–2015; 73%, occurred in patients having a central venous catheter (CVC), 61% with a urinary catheter, 51% had undergone recent surgery, and 41% had diabetes mellitus [14]. Moreover, approximately 41% of patients were prescribed antifungal therapy when *C. auris* was isolated [14]. The median time from admission to infection was 19 days (range 9–36), and 61% of patients had a bloodstream infection. The overall crude in-hospital mortality rate of *C. auris* candidemia ranges from 30 to 60% [6].

Between April 2015 and July 2016, the first European nosocomial outbreak, was described in a London cardio-thoracic center [15], where 50 *C. auris* cases (average age 53 years, range 19–78) were reported. Among them, 44% (22/50) developed possible or proven infection with a 18% (9/50) candidemia rate, also some cases occurred despite treatment with echinocandins.

Moreover, the ECDC reported an outbreak of 33 *C. auris* bloodstream infections that occurred in 2016 in the surgical ICU of a hospital in Spain. One case of *C. auris* isolated from a blood culture was also reported in November 2015 by the German national reference center for invasive fungal infections. Finally, one case of invasive candidiasis, due to an isolate of *C. auris,* was also reported in Norway, but the infection was probably acquired abroad as the patient concerned was transferred from a hospital outside of the European Union [16].

A large majority of *C. auris* isolates are fluconazole resistant (93%), and amphotericin B and echinocandin resistance rates are approximately 30–40% and 5–10%, respectively. Almost half of isolates are MDR (resistant to two or more antifungal classes), and a small percentage are pandrug resistant [17]. Therefore, *C. auris* infections pose a serious challenge regarding identification and therapy, especially in developing countries where modern identification facilities (molecular identification and MALDI-TOF mass-spectrometry) and access to antifungals other than fluconazole are limited.

#### Saprochaete clavata

Several cases of rapidly fatal infections due to the fungus *Saprochaete clavata,* formerly *Geotrichum clavatum*, were reported in France within a short period of time in three health care facilities, suggesting a common source of contamination [18]. Thus, between September 2011 and October 2012, a nationwide alert collected 30 cases of invasive infections in 14 centers, including an outbreak of 18 cases over 8 weeks in 10 health care facilities located in 10 different French regions. The French alert was internationally relayed by the ECDC and CDC but no other country was concerned [19].

Half of the 30 cases reported in the French outbreak were male patients with a median age of 63 years. Most of the patients (70%) were hospitalized for acute myeloid leukemia, were exposed to cytarabine (80%), and all cases received blood products. *S. clavata* was recovered from blood in 87% cases (26/30), from bronchoalveolar fluid or tracheal aspirates in 40% cases (12/30), and 60% of patients had gut colonization. Moreover, diarrhea previous to or associated with fungemia was reported for 61.5% of cases, suggesting the role of a subsequent gut translocation in the occurrence of these bloodstream infections. The case fatality rate at day 60 was 80%, with death occurring at a median of 7 days following diagnosis [18].

Phylogenetic analysis of the French outbreak identified a single clone (clone A) which accounted for 16 of the 18 outbreak cases and that was distinct from sporadic isolates [18]. This finding provides additional, although not definite evidence, for a common source of contamination in this health care facility outbreak. However, epidemiological and microbiological investigations did not lead to a documented source of infection.

Since the 2012 French outbreak, the epidemic clone A of *S. clavata* has been identified several times in France in small clustered or sporadic cases [19]. In Italy, three cases of *G. clavatum* bloodstream infections were reported in one hematological ward in 2016 [20]. All patients received cytarabine and developed *G. clavatum* infection within 3 weeks from therapy initiation. In all cases, visceral localizations (spleen, liver, and lung) were documented by a total body computed tomography scan. A prolonged antifungal therapy with high doses of liposomal amphotericin-B was necessary to obtain fever resolution; one patient did however die.

*S. clavata*, previously reported as *G. clavatum*, had rarely been isolated from human samples before this outbreak [21, 22]. Its ecology, reservoir, and importance in agriculture and food are unknown [23]. This species is closely related to the known human pathogen *Magnusiomyces capitatus* (previously known as *Geotrichum capitatum*) and these two species are often misidentified. Both are resistant to echinocandins, which are recommended in hematology wards in case of candidemia or febrile neutropenia of unknown origin. Sporadic cases and outbreaks of fungemia due to *M. capitatus* have been reported in patients with acute hematological malignancies, and these outbreaks have sometimes been related to contaminated dairy products [18]. In the French *S. clavata* outbreak, results of local investigations of fungal contamination of food, blood products, medical devices, and environmental samples were all negative [18].

#### Sarocladium kiliense

An outbreak of *Sarocladium kiliense*, formerly *Acremonium kiliense*, bloodstream infections occurred from June 2013 to January 2014 and included a cluster of cases at eight hospitals in Santiago, Chile [24]. All 67 patients infected with *S. kiliense* were under chemotherapy and received the same antinausea medication (ondansetron), produced by the same pharmaceutical company in Colombia. Two out of three lots of unopened ondansetron, tested by the Chilean Ministry of Health, yielded vials contaminated with *S. kiliense*, forcing a recall of all ondansetron in Chile made by the Colombian manufacturer. Subsequently, Colombian officials discovered 16 other infected patients who received ondansetron also from the same Colombian pharmaceutical company. Culturing and conventional DNA sequence identification methods confirmed that ondansetron was contaminated with *S. kiliense* [24].

*S. kiliense* has been implicated previously in healthcare-related infections, but the lack of available typing methods has precluded the ability to substantiate sources. The use of WGS-based typing to investigate fungal outbreaks has become integral to epidemiologic investigations. The WGS-based typing analysis demonstrated that the patient isolates from Chile and Colombia were nearly genetically indistinguishable from those recovered from the unopened medication vials, indicating the likely presence of a single-source infection [24].

In 2013 Ioakimidou et al. reported an unusual cluster of possible catheter-related bloodstream infections due to *S. kiliense* (*A. kiliense*) in patients who underwent hematopoietic stem cell transplantation [25]. Three febrile transplanted patients repeatedly yielded the fungus from blood cultures taken through a CVC during a short period of 1.5 months (March to May 2011) in a Hematology Department in Thessaloniki, Greece. The identification of *S. kiliense* was confirmed by sequencing the internal transcribed spacer (ITS) region of three isolates (one from each patient). In accordance with antifungal susceptibility testing, the patients received voriconazole in addition to CVC removal. The outcome was favorable for all the patients.

*S. kiliense*, a hyaline mold formerly known as *Acremonium kiliense*, is a ubiquitous soil saprophyte commonly found in the environment and occasionally infecting humans. Its pathogenicity in immunocompetent patients is low and usually is related to inoculation of the fungus via a penetrating injury that often leads to a granuloma formation. *A. kiliense* has been described as a cause of mycetoma, keratitis, endophthalmitis, endocarditis, continuous ambulatory peritoneal dialysis-associated peritonitis, and catheter-related fungaemia [25]. However, the presence of underlying immunological disorders can predispose to the development of a usually fatal systemic infection such as peritonitis [26].

#### Exserohilum rostratum

In September 2012, the CDC was notified of several cases of meningitis in Tennessee, including one patient with culture-confirmed *Aspergillus fumigatus* meningitis 46 days after an epidural steroid injection. This patient was the index case of the largest fungal infection epidemic in the United States, a public health threat of historically unprecedented magnitude.

Indeed, subsequent investigations revealed a multistate [23] outbreak of fungal infections, primarily *Exserohilum rostratum*, caused by epidural, paraspinal, and peripheral joint injections of fungus-contaminated lots of methylprednisolone acetate (MPA) produced by a single compounding pharmacy [27, 28]. More than 13,000 people were exposed to three lots of contaminated MPA and in October 2013, 751 had developed fungal infections following their MPA injections and resulting in 64 deaths. Specimens from patients were tested for the presence of fungi, and laboratory evidence confirmed the presence of *E. rostratum* in 153 case patients (20%). The fungal infection cases presented a broad spectrum of clinical disease, reflecting the differences in pathogenesis, exposure route, and host risk factors [29]. A total of 233 patients only had meningitis, 325 only had paraspinal/spinal infection, 33 only had peripheral joint infection, 151 had meningitis and paraspinal/spinal infection, two had paraspinal/spinal infection and peripheral joint infection, and seven had a stroke [30].

The overall attack rate was 5.5 cases per 100 exposed persons [28]. Case patients had received a median of one injection (range, 1–6) of implicated MPA lots, with a median age of 64 years (range, 15–97 years), and median incubation period of 48 days (range, 0–249 days) from the last injection to diagnosis [30].

Cohort analysis of patients who had been given epidural or paraspinal glucocorticoid injections in Tennessee showed that the infection risk was higher among patients exposed to: one specific lot among the three contaminated lots, older vials, higher doses, multiple procedures, and in patients in whom a translaminar approach to epidural glucocorticoid injection was used [27]. Tests carried out at CDC and Food and Drug Administration laboratories on the preservative-free MPA vials confirmed the presence of *E. rostratum* in unopened vials from two of the three recalled lots. These laboratory results strengthen the link between the preservative-free MPA vials and the outbreak resulting from a series of sterility assurance failures [31].

The major cause of this outbreak was *E. rostratum*, a dematiaceous (black) mold found in soil and plants debris worldwide, however it is more common in tropical and subtropical areas [27]. This black mold usually infects plants and rarely causes disease in humans. Of the approximately 30 cases of *Exserohilum* infections reported in the literature before this outbreak, the most common presentations were skin, corneal, and sinus infections [29]. Because *Exserohilum* rarely causes human infections, relatively little is known about its physiopathology and management, particularly in the case of central nervous system (CNS) infections. How the pathogen entered the CNS, in this outbreak, is unclear. It may be rarely by direct inoculation of the contaminated material into the subarachnoid space by inadvertent puncture of the dura during the glucocorticoid injection, or more likely, by direct contiguous spread from the site of injection.

### Whole-genome sequence typing: A New molecular tool for uncommon fungal outbreak investigations

Whole-genome sequence (WGS) typing is a new molecular approach that enables genotyping of any microorganism without genetic insights or prior knowledge of the natural population diversity in that species. Thus, it can be used when no other conventional method is available for molecular genotyping. Indeed, when an outbreak is caused by an uncommon fungal species there is usually little epidemiological information available regarding dissemination, genetic diversity, and/or population structure for the species; standard methods for molecular genotyping are often not suitable and lack comprehensive resolution. For these unusual fungal pathogens, WGS typing can be highly valuable for allowing first molecular comparisons of strains [32]. WGS typing is based on the analysis of whole single-nucleotide polymorphisms (SNPs) within each genome, which allows investigating genome-wide variation between isolates. The relationships between the isolates can then be inferred from the number of SNPs differing between them. SNP distances determine genetic relatedness. Another advantage of WGS typing is that it permits the production of “de novo assembly” for these unusual pathogens lacking published reference genomes. Whole-genome sequencing is also useful for getting information characterized important traits, such as the level of ploidy, the size of assembled genome, etc.

WGS typing has a high discriminatory power to relate strains. However, its current limitation is that it must be performed in a laboratory in which the different steps of the process are well established, including sequence acquisition and bioinformatics for handling and analysing the data [33]. Considerable challenges remain due to the lack of common standards, particularly for investigating fungal genomes because they are much larger than those of bacteria and viruses and have a variable level of ploidy.

Recent years have been rich in large fungal outbreaks caused by very unusual fungal species (Table 1). These outbreaks, due to either yeasts or mold, have signaled the beginning of a new era of fungal outbreak investigation based on the comparison of the whole genomes of outbreak strains by using WGS. WGS typing permits investigation into several fungal outbreaks due to uncommon fungal species allowing researchers to decipher epidemiological clues [34–36].

The largest fungal outbreak of healthcare-associated infections investigated using WGS typing was caused by the black soil mold *E. rostratum*, responsible for a number of cases of meningitis in the USA. Owing to epidemiological investigations, the use of MPA injections contaminated with *E. rostratum* was quickly demonstrated to be responsible for the outbreak [37, 38]. Different lots of MPA from a single compounding pharmacy were positive with *E. rostratum*. However, it was not possible to demonstrate the genetic relationship between the fungal isolates due to the lack of relevant typing methods for that given species. WGS typing was eventually used to analyse and compare the genomes of both the clinical isolates and those from MPA vials originating from different lots [39]. It initially demonstrated that the isolates were highly clonal, strongly suggesting a single common source. Secondly, it demonstrated that clinical isolates could not be distinguished from those from the MPA vials, confirming that MPA was the source of infection. Interestingly, WGS analysis also showed that these *E. rostratum* isolates were haploid and nearly identical, only differing by less than 2 SNPs between any pair of isolates. In contrast, hundreds of thousands of SNPs differentiated the isolates responsible for the outbreak and unrelated control isolates. These huge differences between unrelated and related genome strains unearths interesting questions and may indeed suggest that the unrelated control strains belonged to different cryptic species. Nonetheless, this study highlighted the power of the WGS typing to provide accurate information about the relatedness of the isolates of rare species, here *E. rostratum,* when no genetic information was available.

The same approach was then used to investigate the healthcare-associated outbreak of *S. kiliense* bloodstream infections associated with the administration of contaminated antinausea medication among oncology patients in Colombia and Chile [24]. Once again, WGS typing confirmed a common source of infections in the two countries related to the contaminated medication. Indeed, genomes of isolates from infected patients of the two countries and from the different lots of contaminated medication were nearly indistinguishable from one another. No more than 5 SNPs were detected between any pair of isolates; for reference the genome size of *S. kiliense* is approximately 36 MB.

In France, *S. clavata*, another previously unrecognized fungal pathogen, was implicated for different clusters of infections in leukemic patients [18]. The use of WGS typing identified a single clone for most cases. However, it was not possible to connect it to a common source of infection.

An interesting secondary outcome of these three studies was that a large panel of assembled genomes from the three species was produced for the first time. Some of them were from related strains, including genomes from the outbreak strains, while others were from unrelated control strains, from the same or different countries. These genome sequences served as the basis for constructing databases to study genetic variations in these unusual species, which had never been done before. Thus, in addition to their power for genotyping, WGS data generated during outbreak investigations can also to be used to better understand the biology, the virulence, and even possible therapeutic targets in these species.

Recently, the nearly simultaneous global emergence of a novel species of *Candida*, *C. auris*, capable of causing hospital-acquired multidrug-resistant infections, has likely represented one of the more complex challenges in terms of epidemiological investigation. In 2009, when *C. auris* was first isolated from the external ear canal in a Japanese hospital patient, no information about this species or its putative reservoir was available. Since the publication of the first *C. auris* genome sequence in 2015 [40], a large number of *C. auris* strains have been sequenced for investigating different outbreaks. Their analysis suggests that the pandemic is due to recent and independent emergences of different clonal populations of *C. auris*, rather than to the worldwide spread of a dominant clone. Indeed, phylogenetic analysis of the whole genome SNPs from strains isolated from three continents showed that there is a strong phylogeographic structure, with the strains grouping into unique clades by geographic regions [14]. The strains from the same geographic region, such as South Asia, South America, or South Africa, were highly clonal and belonged to a specific clade, with very few SNPs differences (16-70 SNPs). In contrast, tens of thousands of SNPs differentiated strains from different continents.

In addition, recent investigations showed that in a given country, strains isolated from patients hospitalized in different hospitals or towns can be virtually identical (only a few SNP differences) allowing precise tracking of clonal spreading in the different clusters. Finally, a recent study showed almost identical (less than 5 SNP differences) *C. auris* isolates from the surface environment of one hospitalized patient and his own isolates, which further suggested that the spread within health care settings was possible [41]. Overall, WGS exclusively provided unambiguous data on the genome sequences and on the number of SNPs differentiating isolates. Those can easily be shared between laboratories but also centralized for a global investigation of the pandemic [42]. As mentioned above for other uncommon species, these data have also been used to further study genetics of the biology and the virulence of *C. auris* [43].

### From infection control point of view

The various fungal outbreaks described within the last 6 years have highlighted not only the importance of general infection prevention measures but also the importance of warning alert systems. Indeed, the specificity of these outbreaks emphasizes the value of monitoring systems. Concerning *C. auris,* the risk of misidentification was high and it has been suggested that *C. auris* should be suspected in several situations, such as: a) when identification cannot be obtained using traditional biochemical mycological methods, and b) when resistance to more than one antifungal drug is detected for an isolate with an ambiguous identification [44]. Furthermore, concerning the other described species such as *S. clavata, E. rostratum*, and *S. kiliense*, the published studies highlighted several points as the common source of contamination (*E. rostratum* and *S. kiliense*) and the specific populations at risk. It seems fundamental to introduce warning alert systems that could help practitioners and health authorities to react quickly to prepare in the event of an outbreak. These warning alerts could be implemented in specific populations e.g., immunosuppressed patients.

Given the risk of nosocomial transmission of *C. auris*, it is necessary to promptly implement infection control measures to limit its spread. Similar to other *Candida* species, colonized and infected *C. auris* patients share the same risk factors, including diabetes mellitus, abdominal surgery, broad-spectrum antibiotics, and presence of CVCs [17]. Healthcare acquired infections typically occur several weeks (10–50 days) into a patient’s hospital stay [6].

Hand transmission and persistent contamination of environmental surfaces within healthcare facilities have been associated with outbreaks; thus, CDC recommendations are based on standard and contact precautions [45]. As for other *Candida* species, transmission could occur through healthcare workers (HCW) hands; however unlike others HCW seem not to be colonized long term. Indeed, as suggested by two different studies, carriage seems to be rare. The most recent study [46] highlighted that during an outbreak only four among 145 HCW were hand carriers. However systematic sampling of the hands, nose, axilla, groin, and throat of 258 HCW conducted as part of the UK investigation identified only a single HCW with a positive nares swab, who later tested negative from the same site, suggesting transient carriage [15].

All hospitalized patients with *C. auris* infection or colonization should be housed in single rooms with environmental precautions. During outbreak investigations, *C auris* has been isolated from medical equipment likely originating from patient’s skin shedding [47]. Explaining why considerable attention has focused on the efficacy of disinfectants used for skin decolonization and environmental decontamination.

Indeed, several authors suggested that contaminated surfaces in healthcare facilities may be an important source of acquisition [48–50]. Experimentally, *C. auris* and other *Candida* species persisted for 7 days or up to 4 weeks on moist and dry surfaces, respectively [51]. Moreover, *C. auris* exhibited a greater propensity to survive on surfaces than *C. albicans*, but not *C. parapsilosis* or *C. glabrata.* In comparison to common bacterial pathogens, *Candida* species were recovered with similar frequencies from dry surfaces and were recovered significantly more often from moist areas such as sinks. Moreover, *C. auris* has the capacity to form antifungal resistant biofilms sensitive to the disinfectant chlorhexidine in vitro [52]. Correspondingly, during an outbreak in London, Schelenz et al. [15] implemented extreme environmental decontamination measures for cleaning and disinfection of the patient rooms and equipment using 1000 ppm chlorine-based products three times a day. On discharge or transfer of a *C. auris* positive patient, the room was subjected to terminal cleaning with 10,000 ppm chlorine-based detergent and all cleaned equipment was left in the room to be disinfected with hydrogen peroxide vapour.

The CDC recommends thorough daily and terminal disinfection of room surfaces and shared medical equipment in rooms of patients with *C. auris* infection (https://www.cdc.gov/fungal/diseases/candidiasis/c-auris-infection-control.html). Although many disinfectants are registered as disinfectants against *Candida* species by the Environmental Protection Agency, it is recommended that a disinfectant effective against *Clostridium difficile* spores should be used, such as chlorine-based products.

Several recent studies evaluated the efficacy in vitro of disinfectants used for skin decolonization and environmental decontamination during hospital outbreaks [53–55]. All showed differences between products; activity also varied with different formulations. Indeed, chlorhexidine gluconate, iodinated povidone, chlorine and H_2_O_2_ vapour demonstrated effective killing activity against *C. auris* when used in clinical practice. However, among disinfectants utilised for skin decolonization, chlorhexidine was much less inhibitory at 3 min contact time compared to iodinate povidone used at 10% [54]. Similarly, the widely used quaternary ammonium disinfectants had a relatively poor activity against all *Candida* species [55].

Limiting the spread of *C. auris* needs rapid multifaceted interventions (Table 2) including contact isolation, enhancing environmental disinfection, screening contact populations, and skin decolonization for colonized patients. However, there is uncertainty as to how best monitor prolonged colonization (https://www.cdc.gov/fungal/diseases/candidiasis/c-auris-infection-control.html). There are no clear data on the efficacy of decolonization measures for patients colonized with *C. auris*, although chlorhexidine has been tested for that purpose during outbreaks [15, 49].

**Table 2:** Interventions needed in case of *Candida auris* outbreak

Interventions proposed	Usefulness
1-Notify public health agency	Undoubted
2-Place patient (colonized or infected) in a single room	Undoubted
3-Institute Contact Precautions for colonized or infected patients	Undoubted
4-Screen all contact patients (defined as roommates) once a week and before leaving the medical ward	Uncertainty how best to monitor
5-Reinforce environmental cleaning 3× day with 1000 ppm chlorine based, vaporized H2O2	Undoubted
6-Reduce duration of invasive procedures in colonized patients	Undoubted
7-Skin decolonization (colonized patients) with 10% *w*/w iodinated povidone	No clear data

## Conclusions

Currently, invasive fungal infections have undoubtedly been identified as having a major negative impact on human health. Indeed, they are a real threat during the hospital course of the weakest. Data from epidemiological studies has allowed better identification of patients at risk and major prevention measures have been evaluated to decrease the global burden of fungal infections. However, when a new situation occurs, such as a healthcare outbreak due to a fungal species, it is difficult to evaluate which are the most appropriate control measures to implement rapidly, in part, because healthcare fungal outbreaks are rare phenomena. Nevertheless, during recent years we have suffered from four large healthcare fungal outbreaks due to uncommon and new species of fungi, which have been responsible for hundreds of infection cases. The analysis of these rare epidemic phenomena is a unique opportunity to learn how to rapidly identify the first cases, to implement adequate control measures even when published data are lacking, and to reinforce the awareness of the public health community with regards to this new fungal risk.

## Abbreviations

CDC: Center for disease prevention and control
CVC: Central venous catheter
ECDC: European center for disease prevention and control
HCW: Healthcare workers
ICU: Intensive care unit
MALDI-TOF: Matrix-assisted laser desorption ionization-time of flight
MDR: Multidrug-resistant
MPA: Methylprednisolone acetate
WGS: Whole-genome sequencing
WGST: Whole-genome sequence-based typing
